# The global state, trajectory and trends of bacterial extracellular vesicles: a bibliometric and visualized analysis

**DOI:** 10.3389/fmicb.2025.1585809

**Published:** 2025-09-16

**Authors:** Haokai Chen, Qiyu Xie, Jiayu Cai, Qing Xin, Wei Yao, Hong Xu, Zongke Zhou

**Affiliations:** ^1^Department of Orthopedic Surgery and Orthopedic Research Institution, West China Hospital, Sichuan University, Chengdu, China; ^2^West China School of Medicine, Sichuan University, Chengdu, China; ^3^Department of Clinical Medicine, The Third School of Clinical Medicine, Guangzhou Medical University, Guangzhou, China; ^4^Department of Radiation Oncology, Cancer Center, West China Hospital, Sichuan University, Chengdu, China

**Keywords:** bibliometric, bacterial extracellular vesicles, outer membrane vesicles, drug delivery, immunotherapy, nano-materials

## Abstract

**Introduction:**

Bacterial extracellular vesicles (BEVs) are nanoscale biological vesicles secreted by bacteria that carry unique cargo and membrane structures derived from their parental bacteria. In recent years, BEVs have been shown to significantly contribute to the pathogenesis and progression of various diseases, with promising potential as immunotherapeutic agents, drug delivery systems, and nano-vaccines.

**Methods:**

This study aims to comprehensively evaluate the research status and development trends of BEVs using bibliometric approaches. A total of 2,836 publications indexed in the Web of Science Core Collection by 30 November 2024 were included in this study. The R package “Bibliometric” and CiteSpace software were employed for bibliometric analysis and visualization of authors, countries/regions, institutions, journals, subject categories, keywords, and references.

**Results:**

From 2006 to 2024, the number of publications exhibited a steady upward trend, with an annual growth rate of 15.01%. The USA and the Chinese Academy of Sciences were identified as the most productive country and institution, respectively. Gho, Yong Song emerged as the most prolific and influential researcher. Cluster analysis of references revealed that outer membrane vesicles, immunotherapy and probiotics are the three largest research clusters. Keyword burst detection identified cancer immunotherapy, the tumor microenvironment, and drug delivery as promising research directions.

**Conclusion:**

Our study highlights that BEV-based drug delivery systems are a major focus of current research, with tremendous potential. Future research on BEVs is expected to focus on tumor immunotherapies and remodeling of the tumor immune microenvironment. With the advancements in nanotechnology, biomedicine, and industry, BEVs are anticipated to make remarkable strides and greatly contribute to solving challenging clinical issues.

## 1 Introduction

Bacterial extracellular vesicles (BEVs) are nanoscale lipid bilayer vesicles that are typically composed of membrane proteins, lipoteichoic acid or lipopolysaccharides, peptidoglycans, enzymes, and various cargos encapsulated within (Toyofuku et al., 2023). In recent years, the role of BEVs in disease diagnosis and treatment, along with their potential as nanoplatforms, has garnered significant research interest with the increasing understanding of microbial communities in health and disease, as well as the growing knowledge of the role of BEVs in microbial–host interactions. BEVs ranging from 20 to 500 nm in diameter can be secreted by both gram-negative and gram-positive bacteria (Aytar Çelik et al., 2022). The differences in parental bacteria determine the diverse biogenesis mechanisms, unique membrane structures, and varying loading contents of BEVs. BEVs originating from gram-negative bacteria are commonly referred to as outer membrane vesicles (OMVs) or outer-inner membrane vesicles, whereas BEVs derived from gram-positive bacteria are normally known as cytoplasmic membrane vesicles (CMVs) (Toyofuku et al., 2019). BEVs can carry a variety of cargoes from parent bacteria, including lipopolysaccharides (LPSs), nucleic acids, endotoxins, signaling molecules, cytosolic proteins, membrane proteins and peptidoglycan, and perform signal transduction, immune regulation in subsequent ligand–receptor interactions and host cell internalization (Schwechheimer and Kuehn, 2015; Hu et al., 2022).

BEVs reportedly function in the pathogenesis and progression of diseases such as colitis (Wei et al., 2023), systemic bone loss (Kim et al., 2021), diabetes (Seyama et al., 2020) and cardiovascular diseases (Farrugia et al., 2020). On this basis, BEVs have been preliminarily explored as novel diagnostic markers, and targeting BEVs generation and activity has emerged as a promising strategy for treating bacterial infections and related diseases. Given the multiple pathogen-associated molecular patterns and bacterial membrane antigens inherited from their parental bacteria, the potential of BEVs as bacterial vaccines has been extensively explored (Peng et al., 2024). The first OMV-based vaccine was approved in Cuba in 1987 and later licensed in New Zealand, where it successfully controlled the *Neisseria meningitidis* serogroup B (MenB) outbreak (Xie et al., 2022). In addition, BEVs contain numerous immunostimulatory molecules, highlighting their potential as immunotherapeutic agents (Nagakubo et al., 2019). In particular, nanoscale BEVs with hollow structures and natural membrane stability are at the forefront of the development of novel drug delivery systems (Liu et al., 2022). Loaded with therapeutic genetic tools, bioactive molecules, functional materials, and small-molecule drugs, BEVs enable drug targeting to disease regions by decorating the shell with targeting molecules to increase drug accumulation and prevent degradation in the complex physiological environment during transportation (Herrmann et al., 2021). Notably, BEVs exhibit affinity for their parental bacteria due to their similar membrane composition, which gives them excellent potential for antibacterial and biofilm inhibition (Toyofuku et al., 2023; Huang et al., 2022). Compared with mammalian extracellular vesicles (e.g., exosomes), which have garnered significant attention, BEVs present a myriad of unparalleled advantages for the development of drug delivery systems and the regulation of immunity, including high yields, low cost, highly stable membrane structures, outstanding biological tissue barrier penetration, simpler surface design and genetic engineering modifications, and unique immunogenicity (Ho et al., 2024).

Given their tremendous biomedical potential, publications in the field of BEVs have experienced exponential growth over the past two decades. Numerous high-quality original studies have been published, demonstrating the broad applications of BEVs in nanovaccines, nanodrug delivery systems and tumor immunotherapy. However, not all studies address the current challenges, and there remains a long journey from preclinical studies to clinical trials. Therefore, scientific publications in the field of BEVs must be comprehensively analyzed and thoroughly scrutinized. Although many reviews have addressed the fundamentals, challenges, and applications of BEVs, they often rely on the researcher’s subjective understanding of the field and exhibit a degree of bias. Additionally, the lack of objective visualized data hinders a comprehensive analysis of the current state of research.

In light of these considerations, this study employed bibliometric methods to provide a comprehensive evaluation of the BEV field. Bibliometrics is a method for qualitatively and quantitatively analyzing all publications within a specific field over a defined period and has proven to be an effective tool for studying the status and trajectory of a discipline. This study visualizes the authors, countries/regions, institutions, journals, keywords, and references involved in BEVs-related publications, aiming to show the global distribution of academic outputs, assess the current status, analyze research topic evolution, identify research hotspots, define frontiers, and predict future trends. This effort will deepen the understanding of promising BEVs and guide researchers in advancing BEV frontiers.

## 2 Materials and methods

### 2.1 Data sources and search strategy

**Figure 1:**
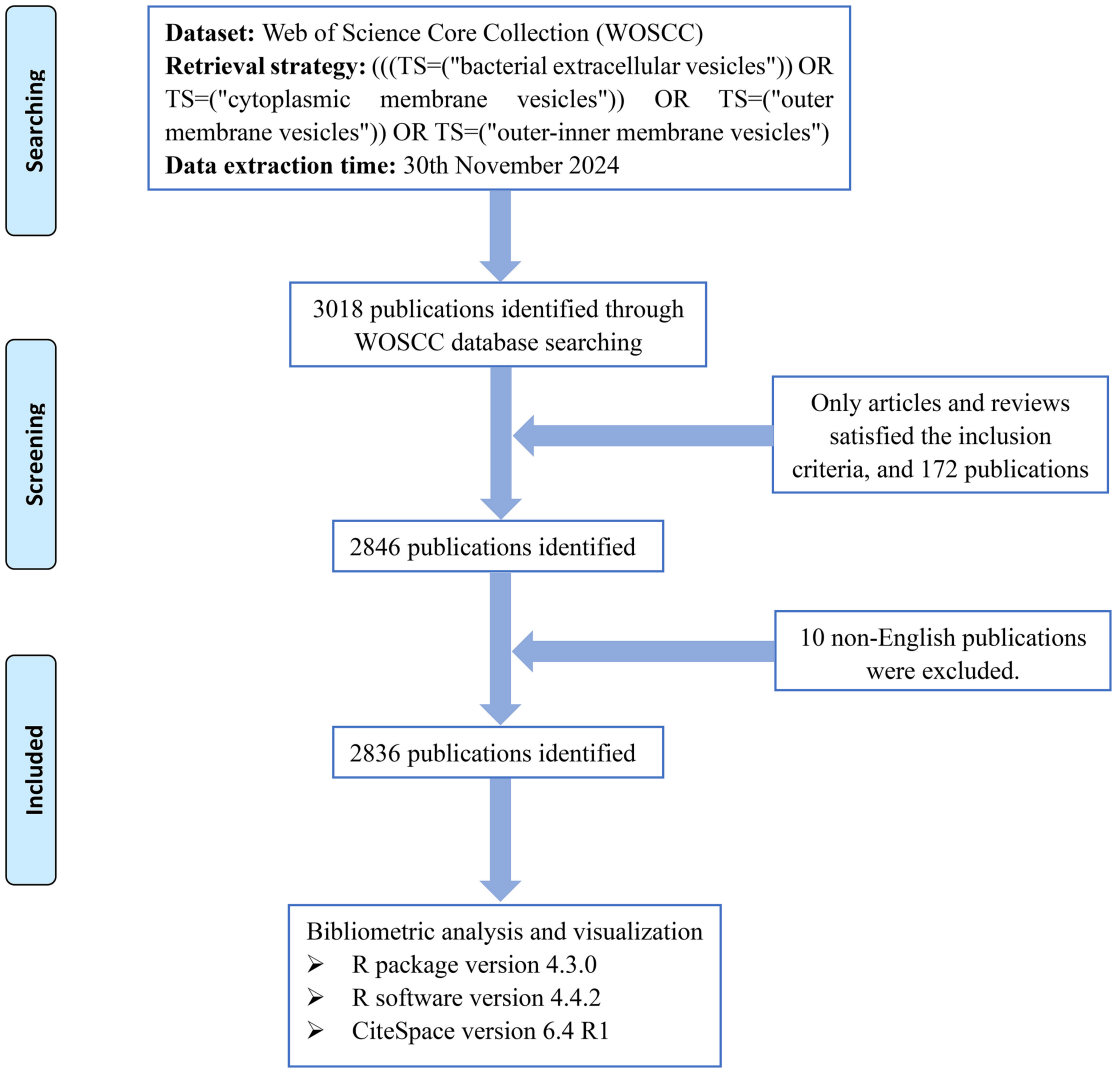
Retrieval and screening flow chart.

For bibliometrics, it is crucial to choose the appropriate database. In this study, the Web of Science Core Collection (WOSCC) dataset stands out for its comprehensive multidisciplinary literature, excellent citation tracking capabilities, and convenient data analyzable formats and was utilized for bibliometric research. The search strategy was as follows: (((TS = (“bacterial extracellular vesicles”)) OR TS = (“cytoplasmic membrane vesicles”)) OR TS = (“outer membrane vesicles”)) OR TS = (“outer-inner membrane vesicles”). The publication or index date was not restricted, and the last retrieval date was 11/30/2024. The type of literature was restricted to research articles and reviews, and the language was limited to English. The 2,836 retrieved documents were subsequently exported in the “plain text file” format, and the content of the records included “full records and cited references.” The comprehensive search and screening process is shown in Figure 1.

### 2.2 Bibliometric analysis

The publication characteristics, including date, author, countries/regions, institutions, keywords, references, journals, and research subjects, were included in the study. The R package “Bibliometric” and CiteSpace software were used for bibliometric analysis and visualization. CiteSpace is a bibliometric tool developed by Prof. Chaomei Chen with comprehensive analysis and excellent visualization capabilities (Chen, 2020). In this study, CiteSpace software was employed for co-occurrence analysis, co-citation analysis, clustering analysis, timeline visualization, burst detection, etc. The R package “Bibliometric” is a reliable tool for bibliometric analysis and has been implemented to map international collaborations, author co-citation networks, and three-field maps.

In the visualized network drawn by CiteSpace, nodes represent specific parameters such as countries/regions, institutions, subjects, cited documents, and keywords. The size of the nodes usually indicates the frequency of occurrence or the number of citations, and the connecting lines between the nodes commonly represent co-occurrence or co-citation strength. The year-wheel-like node is a characteristic of CiteSpace, and its color usually reflects the time of appearance or citation. Betweenness centrality (BC) is a metric that evaluates the importance of a node in a network by counting the number of shortest paths through it, and a node with a BC value greater than 0.1 is considered to be a central node. In CiteSpace software, BC is often used to measure the importance of countries, organizations, keywords, or documents. In addition, modularity (Q value, ranging from 0 to 1) is the indicator that is used to evaluate the modularity of the network, and the clustering results of the network are considered significant when the Q value is > 0.3. The silhouette (S value, ranging from 0 to 1) was utilized as a metric to assess the effectiveness of clustering, and the clustering results were considered highly reliable when the S value was greater than 0.7.

## 3 Results

### 3.1 Overview of scientific outputs

**Figure 2:**
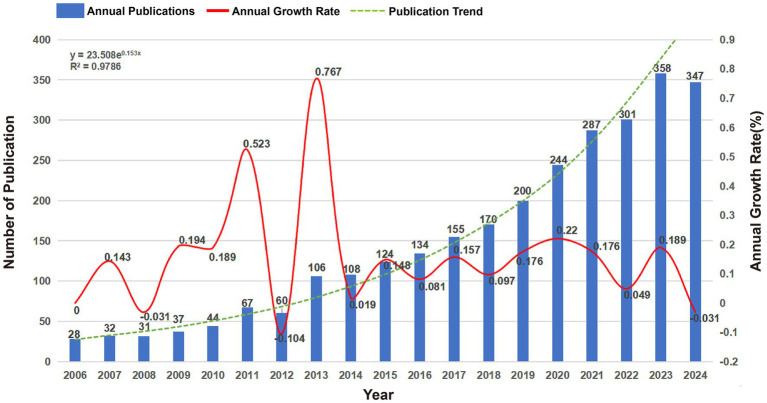
Annual publications, annual growth rates and publication trends of global BEVs from 2006 to 2024.

A total of 2,836 publications related to BEVs sourced from 686 journals spanning the period from 2006 to 2025 were included in this study (three articles published in 2025 were retrieved due to early arrival at WOSCC). A large bibliometric analysis network comprising 13,655 authors, 5,283 author keywords and 125,594 references was constructed. The number of annual publications exceeded 100 for the first time in 2013 and surpassed 300 in 2022 (Figure 2). From 2006 to 2024, the number of publications exhibited a steady upward trend, with an annual growth rate of 15.01% (fitted curve R2 = 0.9786).

### 3.2 Analysis of national/regional contributions

**Figure 3:**
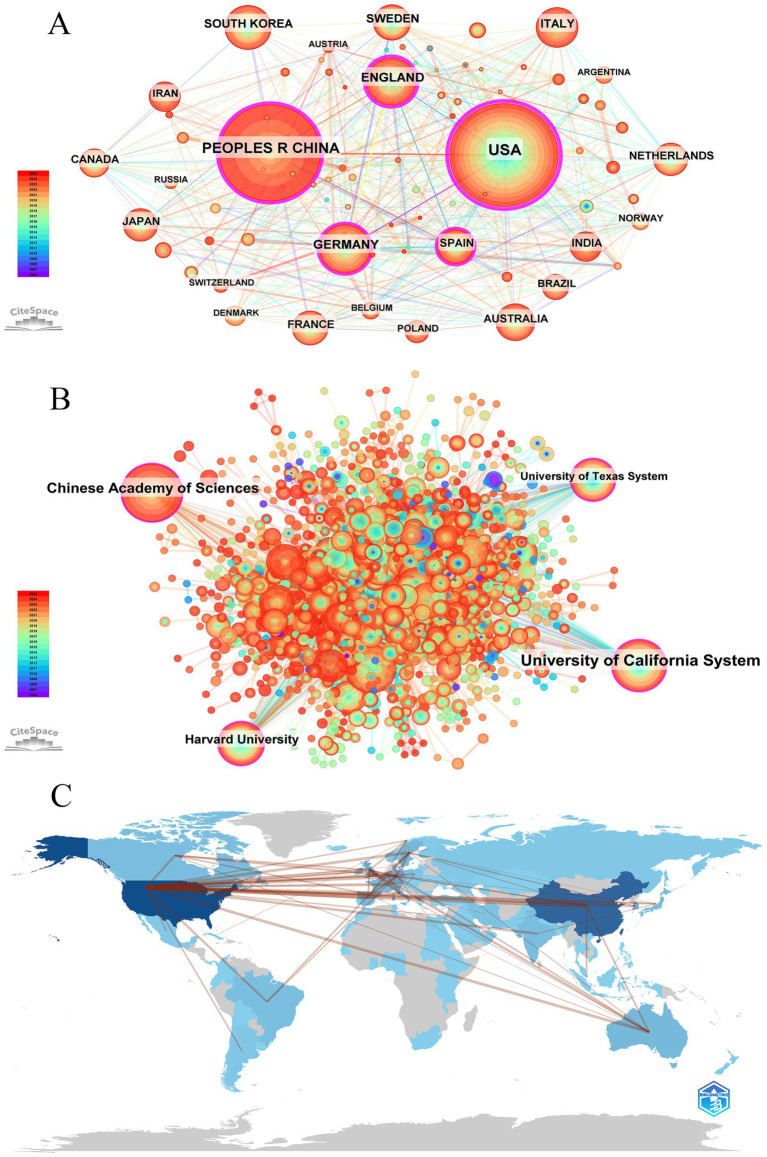
Global visualization of BEVs publications. **(A)** Visualization of the national/regional cooperation network; **(B)** visualization of the global institutional cooperation network; **(C)** world map of international cooperation.

**Figure 4:**
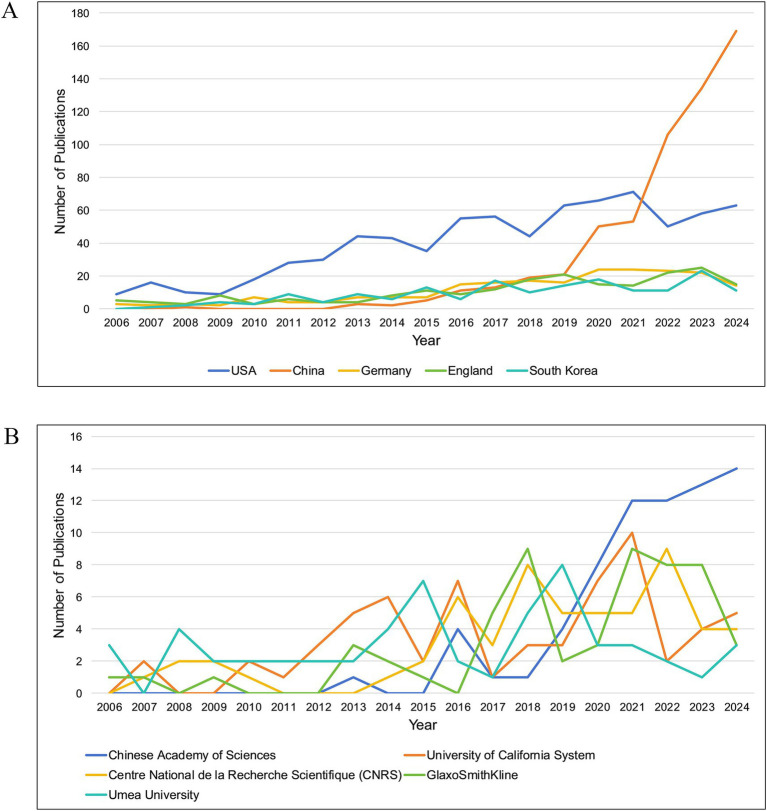
**(A)** The variation in annual publications in the field of BEVs for the top five high-productivity countries/regions; **(B)** The variation in annual publications in the field of BEVs for the top five high-productivity institutions.

Ninety countries and regions contributed to BEV-related research. The top 10 countries in terms of publications are shown in Table 1. The United States (*n* = 768), China (*n* = 588) and Germany (*n* = 217) were the three countries with the most publications. As shown in Figure 3A, the United States, China, Germany, England, and Spain are the five central countries, indicating that they are at the core of international cooperation. Figure 3C shows the world map of international cooperation, clearly showing that China and the USA have the closest cooperation. Figure 4A illustrates the change in annual publications from the top five countries/regions, suggesting that China has made significant investments in BEV-related fields over the past 3 years.

**Table 1:** Top 10 productive countries/regions and institutions in BEVs.

Ranking	Country/region	Count	BC	Ranking	Institution	Count	BC
1	USA	768	0.46	1	Chinese Academy of Sciences	71	0.13
2	CHINA	588	0.23	2	University of California System	63	0.15
3	GERMANY	217	0.13	3	Centre National de la Recherche Scientifique (CNRS)	58	0.08
4	ENGLAND	207	0.22	4	Umea University	56	0.07
5	SOUTH KOREA	172	0	5	GlaxoSmithKline	56	0.04
6	ITALY	160	0.07	6	Helmholtz Association	53	0.06
7	SWEDEN	126	0.06	7	Pasteur Network	51	0.06
8	AUSTRALIA	117	0.03	8	University of Texas System	47	0.10
9	NETHERLANDS	110	0.04	9	Duke University	47	0.02
10	SPAIN	108	0.11	10	Harvard University	43	0.11
10	FRANCE	108	0.08				

Countries/regions and institutions are ranked by the number of publications. BC, Betweenness Centrality.

### 3.3 Analysis of institutional contributions

Over 1,900 institutions worldwide have contributed to BEV-related research. Table 1 shows the 10 institutions with the most publications, with the Chinese Academy of Sciences leading the way with 71 publications, followed by the University of California System (*n* = 63) and Centre National de la Recherche Scientifique (CNRS) (*n* = 58). Four central institutions were identified in the collaboration network: the University of California System, the Chinese Academy of Sciences, Harvard University and the University of Texas System (Figure 3B). Figure 4B illustrates the annual fluctuations in publications from the top five institutions, with the Chinese Academy of Sciences making the most significant progress and maintaining the top position for the last 4 years.

### 3.4 Analysis of authors’ contributions

**Figure 5:**
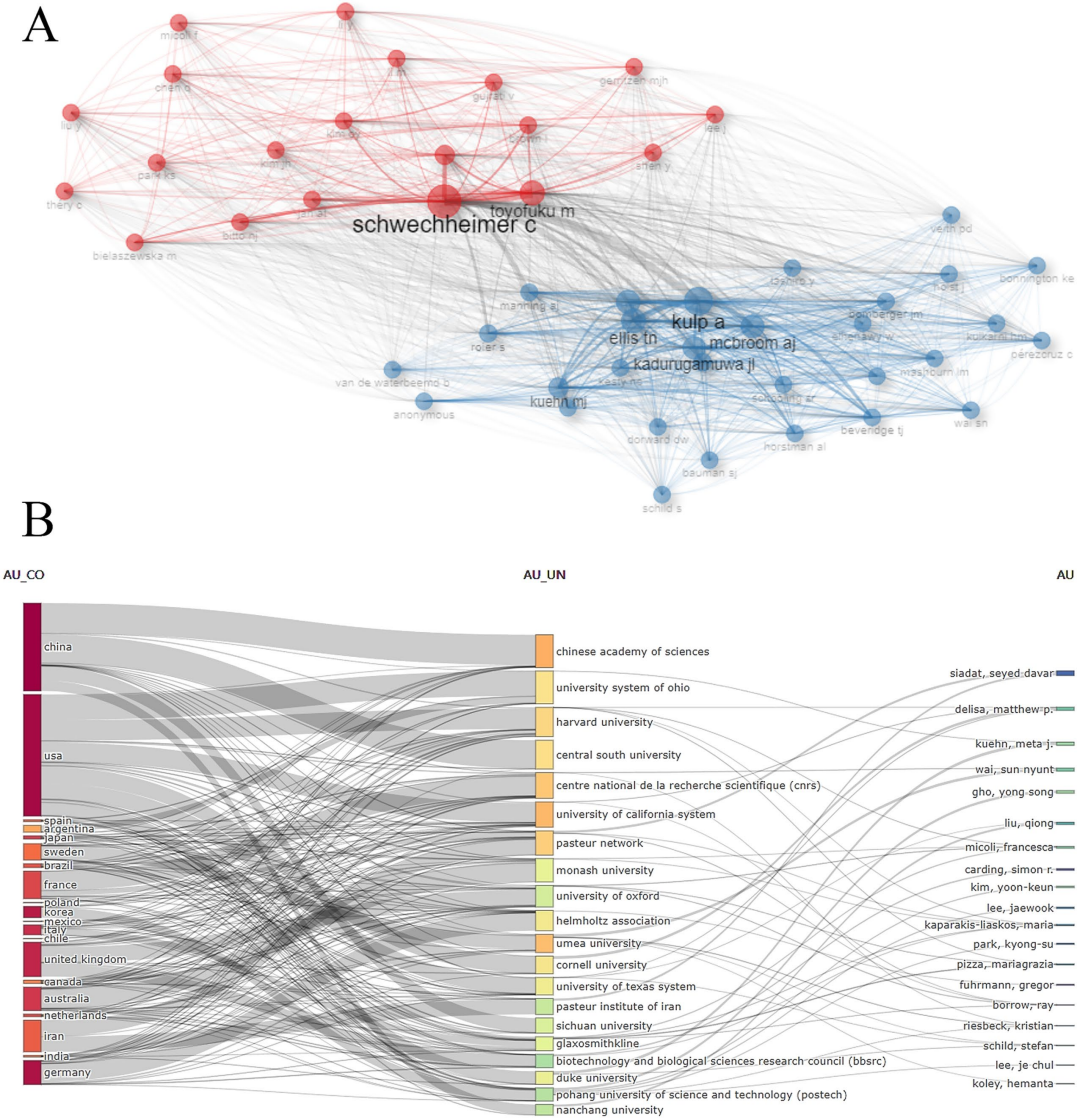
**(A)** Author co-citation network visualization; **(B)** Interconnections between the top 20 most productive countries, institutions and authors.

Over 13,000 researchers have contributed to the field of BEVs. The top 10 authors with the highest productivity are listed in Table 2, where GHO, YONG SONG ranked first with 33 publications, followed by KUEHN, META J (*n* = 32) and KIM, YOON-KEUN (*n* = 29). In this study, all retrieved publications and their references were employed to construct a local knowledge base. KUEHN, META J, GHO, YONG SONG, and FELDMAN, MARIO F are the top three authors with the highest number of local citations. In terms of the H-index, GHO, YONG SONG (*n* = 31), KUEHN, META J (*n* = 25), and WAI, SUN NYUNT (*n* = 22) have the highest local impact. The co-citation network of authors is shown in Figure 5A, where SCHWECHHEIMER and KULP exhibit a high correlation between their research directions. Figure 5B presents the interconnections among the top 20 most productive countries, institutions and authors.

**Table 2:** Top 10 productive authors in BEVs.

Ranking	Author	Count	H-index	Local citations	AC/P
1	GHO, YONG SONG	33	31	1,630	49.39
2	KUEHN, META J	32	25	3,624	113.25
3	KIM, YOON-KEUN	29	19	887	30.59
4	MICOLI, FRANCESCA	26	15	343	13.19
5	WAI, SUN NYUNT	26	22	850	32.69
6	DELISA, MATTHEW P	21	15	556	26.48
7	KOLEY, HEMANTA	20	11	193	9.65
8	FELDMAN, MARIO F	19	17	1,007	53.00
9	LIU, QIONG	19	13	361	19.00
10	SCHILD, STEFAN	19	15	712	37.47

Authors are ranked by the number of publications. AC/P, Average Citations per Publication.

### 3.5 Analysis of journal contributions

BEV-related publications were published in 686 journals worldwide. Table 3 presents the top 10 journals in terms of publications, with FRONT MICROBIOL leading significantly with 144 publications, followed by VACCINE (*n* = 83) and PLOS ONE (*n* = 76). For local citations, INFECT IMMUN (*n* = 9,880), J BACTERIOL (*n* = 7,374) and P NATL ACAD SCI USA (*n* = 5,529) ranked in the top three. In terms of the H-index, FRONT MICROBIOL (*n* = 40), INFECT IMMUN (*n* = 40) and PLOS ONE (*n* = 38) had the highest local impact.

**Table 3:** Top 10 productive journals in BEVs.

Ranking	Journal	Articles	H-index	Local citations	AC/P	JCR	IF (2023)
1	FRONTIERS IN MICROBIOLOGY	144	40	3,905	97.63	Q2	4
2	VACCINE	83	31	5,461	176.16	Q2	4.5
3	PLOS ONE	76	38	5,289	139.18	Q1	2.9
4	INFECTION AND IMMUNITY	70	40	9,880	247.00	Q2	2.9
5	FRONTIERS IN IMMUNOLOGY	68	23	2,101	91.35	Q1	5.7
6	INTERNATIONAL JOURNAL OF MOLECULAR SCIENCES	66	23	1,472	64.00	Q1	4.9
7	SCIENTIFIC REPORTS	57	27	2,917	108.04	Q1	3.8
8	FRONTIERS IN CELLULAR AND INFECTION MICROBIOLOGY	45	20	1,128	56.40	Q1	4.6
9	JOURNAL OF BACTERIOLOGY	43	27	7,374	273.11	Q3	2.7
10	MBIO	36	22	1740	79.09	Q1	5.1

Journals are ranked by the number of publications. AC/P, Average Citations per Publication.

### 3.6 Subject category analysis

**Figure 6:**
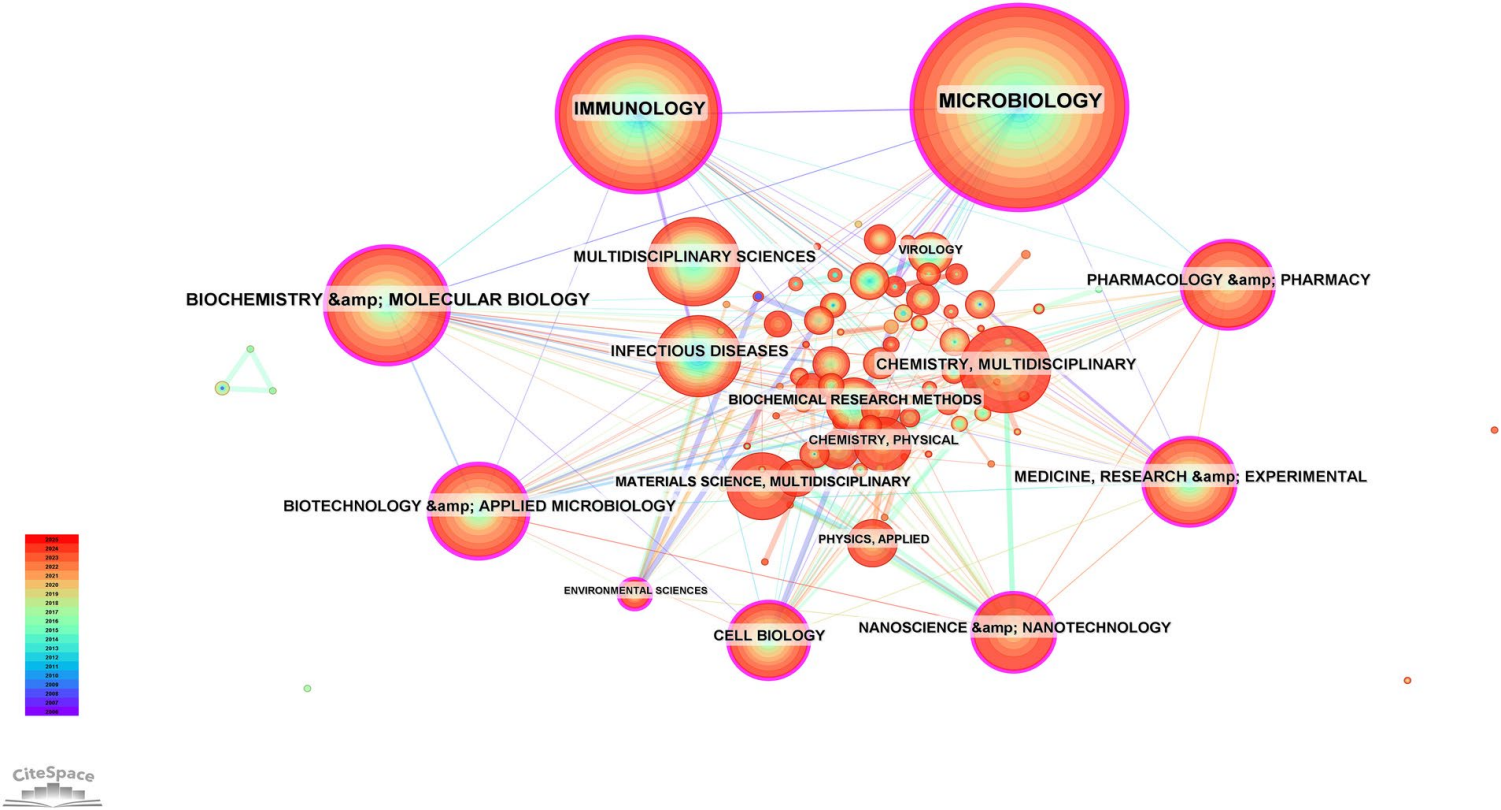
Collaboration network of subject categories.

A total of 101 research categories associated with BEVs were identified through CiteSpace (Figure 6). MICROBIOLOGY (*n* = 941), IMMUNOLOGY (*n* = 589), and BIOCHEMISTRY & MOLECULAR BIOLOGY (*n* = 357) were the three categories with the highest number of publications. Nine center research categories were identified: BIOCHEMISTRY & MOLECULAR BIOLOGY, MICROBIOLOGY, IMMUNOLOGY, BIOTECHNOLOGY & APPLIED MICROBIOLOGY, MEDICINE, RESEARCH & EXPERIMENTAL, CELL BIOLOGY, ENVIRONMENTAL SCIENCES, PHARMACOLOGY & PHARMACY, NANOSCIENCE & NANOTECHNOLOGY. The multidisciplinary categories involved in BEVs suggest significant potential in the interdisciplinary field. Interdisciplinary collaboration facilitates the flow of knowledge from different fields and promotes the advancement and application of BEVs.

### 3.7 Reference analysis

**Figure 7:**
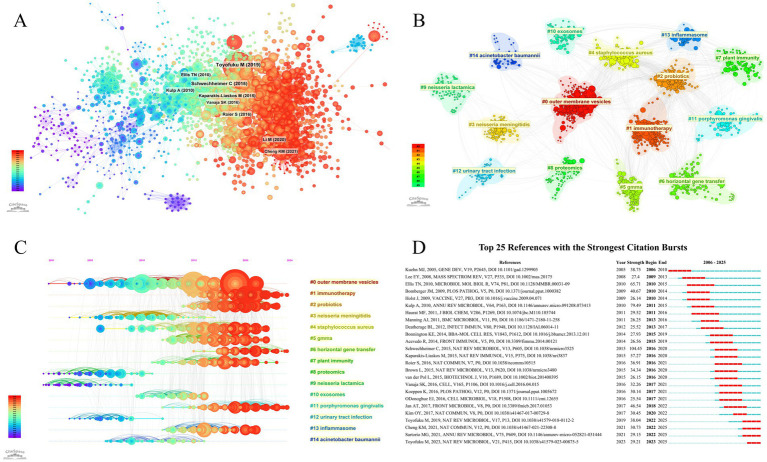
**(A)** References co-citation network; **(B)** Visualization of reference cluster analysis; **(C)** Timeline visualization of reference clustering; **(D)** Top 25 references with the strongest citation bursts.

A total of 125,594 references were identified and employed to construct a comprehensive knowledge base of BEVs and their related fields. The co-citation network of the references was mapped on the basis that the cited documents were simultaneously cited by the citing documents, revealing a strong connection between the references (Figure 7A). Subsequently, 15 clusters were identified through clustering analysis (*Q* = 0.7829, *S* = 0.8853), with outer membrane vesicles, immunotherapy, and probiotics being the three largest clusters (Figure 7B). Furthermore, the timeline visualization of the references illustrates the trajectory of the evolution of studies related to BEVs (Figure 7C). Over time, the research focus has gradually shifted from Neisseria, *Acinetobacter baumannii*, and proteomics to outer membrane vesicles and generalized module of membrane antigens (GMMA) technologies, immunotherapy, probiotics, and horizontal gene transfer.

Burst detection is utilized to capture references with rapidly increasing citations within a given period, which typically have a strong impact on the research field and suggest actively discussed topics within that period. The top 25 references in terms of burst strength are presented in Figure 7D.

### 3.8 Keyword analysis

**Figure 8:**
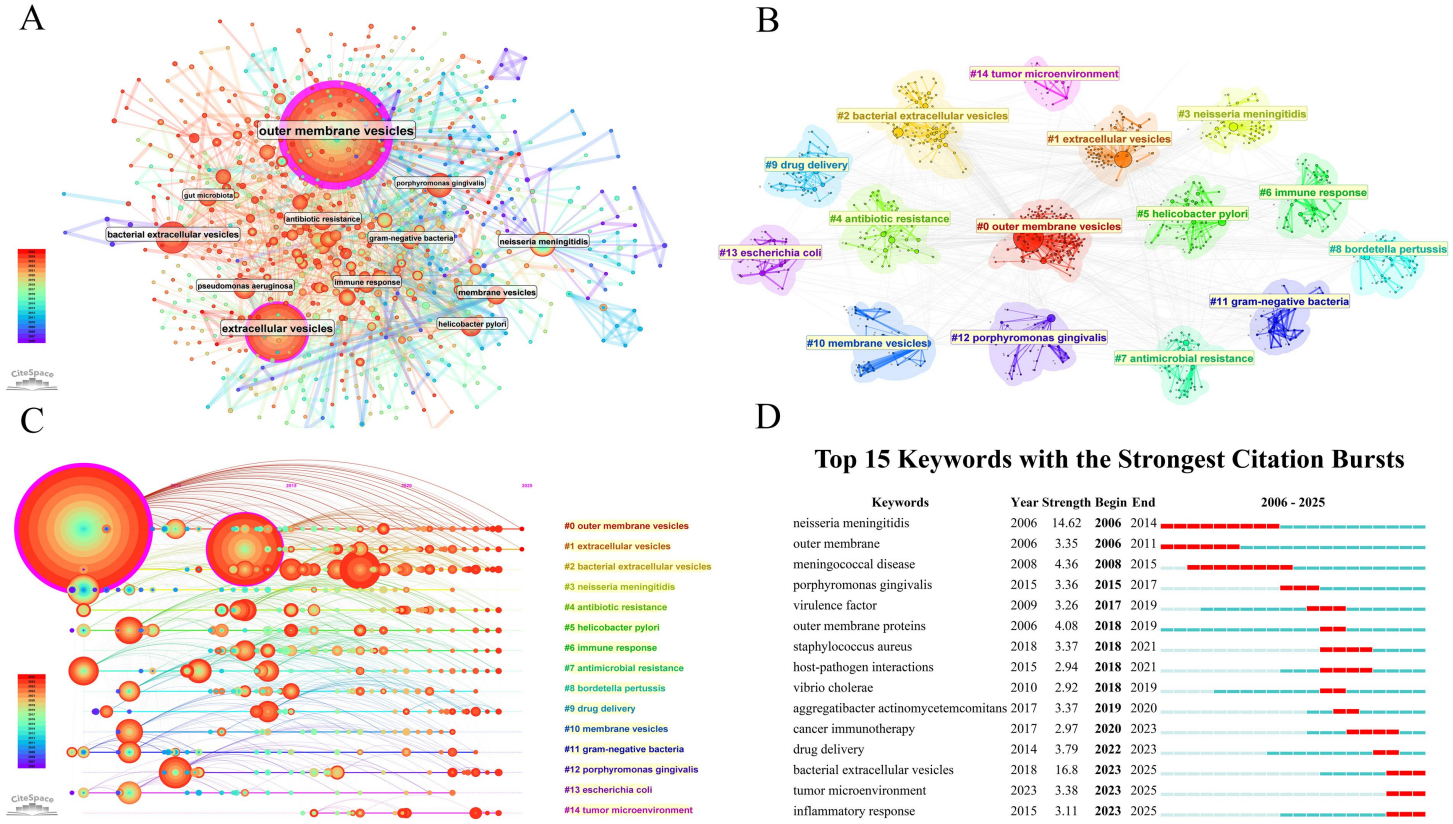
**(A)** Co-occurrence network of keywords, **(B)** visualization of keyword cluster analysis, **(C)** timeline visualization of keyword clustering, and **(D)** top 15 keywords with the strongest citation bursts.

Co-occurrence networks of keywords were constructed, with outer membrane vesicles (BC = 1) and extracellular vesicles (BC = 0.21) identified as central keywords (Figure 8A). Subsequent clustering analysis identified 15 keyword clusters (*Q* = 0.6031, *S* = 0.8987), with outer membrane vesicles, extracellular vesicles, and bacterial extracellular vesicles being the three largest clusters (Figure 8B). Furthermore, the timeline visualization of the keyword clusters demonstrated the research evolution trajectory of BEVs (Figure 8C). Notably, outer membrane vesicles are a type of bacterial extracellular vesicle; however, the concept of extracellular vesicles encompasses bacterial extracellular vesicles but often refers specifically to vesicles secreted by mammalian cells. The evolutionary trajectory of the terms above is indicative of the process of understanding BEVs, starting with the discovery of outer membrane vesicles, followed by a gradual and comprehensive understanding of BEVs. Over time, the research focus has gradually evolved from the bacteria themselves to bacteria–host interactions such as antibiotic resistance and immune responses, as well as applications of BEVs in drug delivery and the tumor microenvironment. In addition, *Porphyromonas gingivalis* and *Helicobacter pylori* have been the most emphasized bacterial types studied in the last 5 years. Keyword burst detection was used to capture keywords whose frequency sharply increased over a specific period, thereby revealing hotspots within that time. The top 15 keywords in terms of burst strength are illustrated in Figure 8D. Cancer immunotherapy, drug delivery, bacterial extracellular vesicles, the tumor microenvironment, and the inflammatory response are the five most recent burst keywords.

## 4 Discussion

BEVs are nanoscale biological vesicles secreted by bacteria that carry unique cargo and membrane structures from their parental bacteria. The understanding of the biogenesis, biological functions and role of BEVs in microbial–host interactions is increasing. BEVs have been shown to significantly contribute to the pathogenesis and progression of various diseases and have considerable potential as immunotherapeutic agents, advanced drug delivery systems, and nanovaccines. BEV-related publications have experienced exponential growth over the past two decades. To the best of our knowledge, this study is the first to analyze the extensive number of publications in the field of BEVs via bibliometric methods.

### 4.1 General information of BEV-related research

A total of 2,836 publications indexed in the WOSCC as of 30 November 2024 were included in this study. Although some studies are still in the process of being published in 2024, the number of publications related to BEVs continues to show a strong upward trend, with a growth rate exceeding 15% from 2006 to 2024. The continued increase in publications emphasizes that BEVs are an emerging, promising and underdeveloped field. BEV research is driven by the collaboration of 90 countries/regions and over 1,900 institutions worldwide. Nine of the top 10 countries in terms of publications are developed, reflecting a direct correlation between economic conditions and biomedical research. China ranks second globally in total publications, following the United States. However, China’s leading publication count and growth rate over the last 5 years suggest that the center of BEV research has gradually shifted from the United States to China. The Chinese Academy of Sciences has the highest number of publications. The number of leading publications in the last 5 years reflects the substantial investment of the institution in BEV research. Consistent with national development, the growth of China’s economy over the past two decades and increasing financial support for medical research have significantly contributed to the progress of China and its institutions. However, the lack of high-impact researchers and publications in China indicates that Chinese researchers need to focus more on the quality of their research in the future. In terms of publication sources, *Frontiers in Microbiology* has published the largest number of articles and holds the highest H-index, reflecting both its strong interest in BEV-related research and its prominent contribution to the field. Multidisciplinary collaboration has played a vital role in driving the advancement of BEV research. Multidisciplinary collaboration has significantly driven the advancement of BEV-related research. The close connections between BEVs and various disciplines underscore their potential as a cross-disciplinary research platform. he central roles of Nanoscience & Nanotechnology, Biotechnology & Applied Microbiology, Pharmacology & Pharmacy, and Medicine within the BEV research network highlight the promising future of BEVs as innovative platforms in nanomedicine, advanced drug delivery, and precision therapeutics.

### 4.2 A challenge journey for BEVs

Research related to BEVs has experienced remarkable progress, increasing from 28 publications in 2006 to over 340 publications in 2024. Various factors, including technology, medical advancements, and major discoveries, influenced the emergence of specific research themes and hotspots at each stage. Burst testing based on references and keywords to analyze research topics, hotspots, and important publications within different stages of development helps researchers understand the trajectory of BEVs, predicts future research directions, and provides valuable insights for researchers in the field.

#### 4.2.1 2006–2015: embryonic stage

A total of 637 publications related to BEVs were published during this phase, accounting for 22.5% of the total. The number of publications fluctuated during this period. *Neisseria meningitidis*, meningococcal disease, and the outer membrane were the burst keywords at this stage, indicating a rapid increase in discussion surrounding OMVs and the development of vaccines for *Neisseria meningitidis*.

*Neisseria meningitidis* is a gram-negative diplococcus that causes invasive meningococcal disease, a serious, life-threatening disease with a fatality rate of 5–15% (Sadarangani and Pollard, 2016). Vaccines against *Neisseria meningitidis* constitute an effective strategy for preventing invasive meningococcal disease and controlling its epidemiology (Piccini et al., 2016). The characteristics of OMVs make them promising platforms for the development of *Neisseria meningitidis* vaccines. Currently, OMV-based vaccines for the prevention of meningitis caused by *Neisseria meningitidis* serogroup B (MenB) are prominent among the few licensed OMV vaccines (Micoli et al., 2024). A review of the *Neisseria meningitidis* OMV vaccine published by Holst et al. (2009) raised significant interest and became a burst reference from 2010 to 2014. This paper summarizes experiences from Cuba, Norway, and New Zealand, demonstrating that the wild-type outer membrane vesicle is the only effective agent against serogroup B meningococcal disease (Sierra et al., 1991). In addition, issues regarding the characterization, manufacture, immune response, efficacy, and safety of the OMV-based *Neisseria meningitidis* vaccine are thoroughly described in this paper, making it an intellectual cornerstone in the field.

Despite the promising clinical utility of OMV-based vaccines in regional MenB outbreaks (Holst et al., 2009; Bjune et al., 1991; O'Hallahan et al., 2009), their immunological limitations became increasingly evident during this period. Most notably, their protective efficacy is predominantly strain-specific, as the immune response is largely directed toward the PorA outer membrane protein, a highly variable and immunodominant antigen (O'Hallahan et al., 2009; Tappero et al., 1999). Consequently, conventional OMV vaccines were insufficient for broader protection across antigenically diverse MenB strains. This inherent limitation underscored an urgent need for more universal meningococcal vaccines and catalyzed mechanistic investigations and bioengineering efforts aimed at expanding the antigenic breadth of OMV formulations.

Encouragingly, several innovative approaches emerged within this timeframe. A promising example was the development of MenPF-1, a recombinant OMV vaccine engineered to constitutively express FetA which is an iron-regulated outer membrane protein known for its immunogenicity and relative sequence conservation (Marsay et al., 2015). A phase I clinical trial demonstrated that MenPF-1 successfully induced bactericidal antibodies against both FetA and PorA, establishing a proof-of-concept for broadening OMV antigenic scope via genetic modification (Marsay et al., 2015). This strategy represented a conceptual evolution from empirical OMV extraction to rational antigen design and manipulation, setting the stage for multi-targeted vesicle-based vaccines.

Simultaneously, another transformative milestone was the development and licensure of the multi-component serogroup B meningococcal vaccine (4CMenB). In contrast to previous single-component OMV vaccines, 4CMenB incorporated OMVs from the New Zealand outbreak strain NZ98/254 alongside three recombinant protein antigens identified through reverse vaccinology (Giuliani et al., 2006; O'Ryan et al., 2014). This vaccine design reflected a paradigm shift, combining the intrinsic adjuvanticity and membrane-bound antigen display of OMVs with the precision of genomics-driven antigen discovery. Two clinical studies published in 2013 further substantiated its safety and broad strain coverage, establishing 4CMenB as a significant breakthrough in meningococcal disease prevention (Vogel et al., 2013; Vesikari et al., 2013). The successful development of OMV-based vaccines has laid the groundwork for elucidating the immunogenic properties of BEVs and has catalyzed broader research into BEV-based nanovaccine platforms.

Insights into the biological functions and genesis of BEVs also developed during this period, with many original and remarkable studies published. Initially, when researchers reported that virulence factors mediate bacterial–host interactions, close contact, such as the type III secretion system (t3SS), was considered essential (Ernst, 2000). As research has progressed, OMVs have been described as a new contact-free secretion mechanism through which bacteria can deliver various proteins into host cells (Kadurugamuwa and Beveridge, 1995; Nguyen et al., 2003). In 2009, Bomberger et al. skillfully designed a study based on *Pseudomonas aeruginosa* OMVs and successfully elucidated the mechanism of long-distance delivery of bacterial virulence factors (Bomberger et al., 2009). OMVs deliver multiple virulence factors simultaneously and directly to the host cytoplasm through fusion with lipid rafts on the host cell membrane and neuronal-WASP-mediated actin transport (Bomberger et al., 2009).

Crucially, the biological functions exerted by BEVs at distant sites are inherently shaped by the selective cargo-sorting mechanisms employed by the parental bacteria during vesicle biogenesis. In 2010, Haurat and colleagues performed an important study using a human oral pathogen, *Porphyromonas gingivalis*, to elucidate the mechanism by which proteins are selectively sorted into OMVs (Haurat et al., 2011). Under the guidance of LPS, gingipains, rather than abundant membrane proteins, are selectively packaged into OMVs (Haurat et al., 2011). Advances in the mechanisms of cargo sorting and long-distance delivery by BEVs have profoundly shaped the subsequent decade of research into host–pathogen interactions. Importantly, these insights extended beyond the context of natural infection, giving rise to a conceptual framework that envisions BEVs as customizable delivery vehicles. Rather than being a passive or stochastic process, cargo incorporation into BEVs has emerged as a regulated and potentially engineerable event. This paradigm shift has opened new avenues for the development of BEV-based drug delivery systems and nanovaccines.

With the increase in the number of antibiotic-resistant bacterial species, the role of OMVs in coping with complex and harsh external environments became a key topic during this period. Many studies have reported the protective effects of OMVs on bacteria. Yonezawa et al. reported that *Helicobacter pylori* OMVs contribute to biofilm formation, which is important for bacterial defense against harsh external environments (Yonezawa et al., 2009). Additionally, McBroom et al. proposed that bacterial OMVs are produced as an envelope stress response for external stress relief (McBroom and Kuehn, 2007). In 2011, Manning et al. revealed that OMVs exert cytoprotective effects by adsorbing external stressors, including antimicrobial peptides and T4 bacteriophages (Manning and Kuehn, 2011). Furthermore, the production of OMVs was shown to be a positive response to antimicrobial peptides (Manning and Kuehn, 2011). Together, these findings highlight the essential role of BEVs in promoting bacterial survival under environmental stress, either by contributing to biofilm formation or by serving as decoys that neutralize harmful agents. This protective function renders BEVs themselves potential targets for antimicrobial intervention. In addition, the ability of probiotic-derived BEVs to compete with pathogenic bacteria for ecological niches and counteract their detrimental effects has stimulated growing interest in their role in maintaining gut microbial homeostasis.

Notably, many reviews on OMVs published during this period had a profound impact on subsequent studies (Kuehn and Kesty, 2005; Lee et al., 2008; Ellis and Kuehn, 2010; Kulp and Kuehn, 2010; Deatherage and Cookson, 2012).

#### 4.2.2 2016–2020: bridge stage

A total of 903 publications were generated in the field of BEVs during this period, accounting for 32% of the total, and the number of publications increased rapidly. Virulence factors, outer membrane proteins, host–pathogen interactions, and bacteria such as *Porphyromonas gingivalis*, *Staphylococcus aureus*, *Aggatibacter actinomycetemcomitans*, and *Vibrio cholerae* were the burst keywords during this period. The biogenesis of OMVs and their role in host–pathogen interactions were further explored. OMVs originating from certain specific bacteria are also discussed in greater detail.

Previously, several models for the formation of OMVs have been proposed, but they were limited to specific bacterial species or required genetic manipulations (Kadurugamuwa and Beveridge, 1995; Kulp and Kuehn, 2010; Kulkarni and Jagannadham, 2014; Haurat et al., 2015). This limitation was overcome by the pioneering work of Roier et al. (2016). The decreased or absent expression of the vacJ and/or yrb genes resulted in the accumulation of phospholipids in the outer leaflet of the outer membrane. The subsequent asymmetric expansion of the outer leaflets leads to outward bulging of the outer membrane. Further accumulation of phospholipids in the leaflet promotes budding, and phospholipids eventually pinch off to form OMVs. This mechanism, which is based on the accumulation of phospholipids in the outer membrane leaflets, represents the general mechanism by which OMVs are produced by gram-negative bacteria (Roier et al., 2016). Further elucidation of the biogenesis of OMV has laid a conceptual foundation for the rational design of strategies to enhance OMV production. By targeting key regulators of outer membrane lipid asymmetry, such as the VacJ/Yrb ABC transport system, it may become possible to manipulate vesiculation rates in a controlled and scalable manner.

The effect of OMVs on host–pathogen interactions is another widely discussed topic during this period. The activation of caspase-11 triggered by lipopolysaccharide is a critical component of host resistance to gram-negative bacterial infection. Initially, it was thought that LPS could be detected only by TLR4 at the cell membrane surface through binding with MD2 and CD14 (Park et al., 2009; Poltorak et al., 1998). As research progressed, LPS was found to enter the cell membrane and stimulate caspase-11. Given that most gram-negative bacteria that activate caspase-11 are not cytosolic, the mechanism by which their LPS enters the cell membrane remains unclear. The work of Vanaja et al. resolves this question (Vanaja et al., 2016). Their study revealed that OMVs act as key carriers to deliver LPS to the cytosol, triggering a caspase-11-mediated response. Specifically, OMVs that enter host cells via clathrin-mediated endocytosis can release LPS into the cytosol from early endosomes, thereby triggering caspase-11-dependent pyroptosis and caspase-1 activation. More broadly, this study reveals a fundamental mechanism by which pathogen-associated molecular patterns (PAMPs) are delivered to the cytosol, where they activate the immune system. Additionally, sRNAs in OMVs are another topic of interest in the context of host–pathogen interactions. Koeppen et al. reported that sRNA52320 contained in *Pseudomonas aeruginosa* OMVs can be transferred to the human airway epithelium, where it inhibits LPS and OMV stimulated IL-8 secretion and neutrophil infiltration (Koeppen et al., 2016). The ability of OMVs to suppress host immune responses through the delivery of specific molecular cargo underscores their multifaceted role in immune modulation. Overall, this period witnessed significant advances in understanding of BEVs as mediators of pathogen–host interactions, particularly in modulating host immune responses. BEVs were increasingly recognized not merely as passive carriers of immunostimulatory molecules, but as active participants that shape host immunity through complex and multifaceted mechanisms. This expanded conceptualization has, to some extent, catalyzed the subsequent development of BEV-based immunotherapeutic strategies.

Several reviews on the biogenesis and function of OMVs, immunomodulation, their potential as vaccines, and mechanisms of entry into host cells have received burst citations, suggesting that the issues discussed in these reviews have gained rapidly increasing interest during this period (Schwechheimer and Kuehn, 2015; Bonnington and Kuehn, 2014; Acevedo et al., 2014; Kaparakis-Liaskos and Ferrero, 2015; Brown et al., 2015; van der Pol et al., 2015; O'Donoghue and Krachler, 2016; Jan, 2017).

#### 4.2.3 2021-present: applications and future

A total of 1,293 publications, accounting for 45.6%, have been published in the field of BEVs from 2021 to the present. The large number of publications during this period reflects the prosperous development of BEVs over the past 5 years. Cancer immunotherapy, drug delivery, bacterial extracellular vesicles, the tumor microenvironment, and the inflammatory response were the burst keywords of this period. Notably, the burst strength of the tumor microenvironment has persisted, suggesting that the application of BEVs in the tumor microenvironment is a promising research direction for the future.

##### 4.2.3.1 BEVs and drug delivery systems

Nanoscale BEVs with natural membrane stability and low immunogenicity are promising candidates for advanced drug delivery systems. To the best of our knowledge, the concept of BEVs as drug delivery systems was not formally proposed until 2014. In this year, Gujrati et al. described OMVs loaded with small interfering RNA (siRNA) targeting the kinesin spindle protein, which could lead to gene silencing and induce tumor regression (Gujrati et al., 2014). However, BEV-based drug delivery systems were not fully developed during the subsequent 5 years. Although some excellent reviews have proposed prospective ideas, few original studies have been published (Kim et al., 2015; Alves et al., 2015; Toyofuku et al., 2015; Watanabe, 2016; Jain and Pillai, 2017; Farjadian et al., 2018). In 2019, research by Carvalho et al. revived the potential of BEVs as drug delivery systems. They engineered *Bacteroides thetaiotaomicron* (Bt) and encapsulated proteins from multiple sources in its OMVs. Bt-OMVs were subsequently shown to package antigens, act as vaccines or express keratinocyte growth factor-2 to promote intestinal epithelial repair in animals (Carvalho et al., 2019). In 2020, Huang et al. designed antibiotic-loaded OMVs that effectively entered and killed pathogenic bacteria (Huang et al., 2020). In another study, Kuerban et al. constructed a DOX-OMV by encapsulating the broad-spectrum antineoplastic agent doxorubicin (DOX) in attenuated *Klebsiella pneumonia*-secreted OMVs. This DOX-OMV significantly inhibited tumor growth in non-small cell lung cancer and recruited macrophages to the tumor microenvironment due to its immunogenicity (Kuerban et al., 2020). Since 2022, the field received unprecedented attention, as several excellent reviews concurrently demonstrated the remarkable potential of BEVs in nanomedicine delivery systems (Xie et al., 2022; Liu et al., 2022; Huang et al., 2022; Ding et al., 2022; Imran et al., 2022; Gao et al., 2022; Kong et al., 2023). The potential of BEVs in advanced drug delivery systems has been further explored, with a growing number of sophisticated strategies being devised to enhance delivery specificity and achieve controlled release of therapeutic cargos.

On the basis of the pathogen-associated molecular patterns derived from parental bacteria on the surface of OMVs, enhancing the penetration and targeting ability of OMVs through hitchhiking of immune cells is a useful natural trafficking strategy. By hitchhiking on neutrophils, Mi et al. designed doxorubicin (DOX)-loaded OMVs that could efficiently cross the blood–brain barrier (BBB) and accumulate in tumors (Mi et al., 2023). Similarly, Pan et al. proposed a new therapeutic strategy for ischemic stroke by encapsulating pioglitazone in OMVs and enhancing the delivery of pioglitazone to the brain via neutrophil hitchhiking (Pan et al., 2023). More recently, immune cell–mediated delivery has been further refined in a spatiotemporally targeted delivery system tailored to the dynamic pathophysiology of spinal cord injury. In an innovative study, researchers developed biomimetic bacterial outer membrane nanoparticles (BM-NPs) by fusing detoxified OMVs (dOMVs) with liposomes co-loaded with rapamycin (Rapa) and LXR-623 (Li et al., 2025). This hybrid nanoparticles strategically capitalized on neutrophil-mediated rapid delivery during the acute phase and macrophage-mediated sustained release during the subacute phase, an approach metaphorically described as the “Tortoise and Hare” model (Li et al., 2025). The results showed that BM-NPs not only suppressed lipid metabolic dysregulation in the injured spinal cord but also preserved myelin integrity and significantly promoted neurological recovery (Li et al., 2025). The fusion of BEVs with synthetic nanocarriers represents an efficient delivery platform design, combining the precise targeting capabilities conferred by BEV membrane proteins with the high drug-loading capacity and enhanced physicochemical stability of synthetic vectors. Importantly, the design of temporally responsive delivery systems, based on the dynamic infiltration patterns of immune cells at different disease stages, offers a promising direction for achieving precision medicine in conditions characterized by evolving pathological microenvironments.

An emerging avenue in the development of BEV-based delivery systems lies in their integration with physical therapies, offering a promising multimodal strategy for cancer treatment. Guan et al. stabilized the photosensitizer chlorin e6 (Ce6) within the phospholipid bilayer of engineered OMVs loaded with CD47 via hydrophobic interactions (Guan et al., 2025). These multifunctional nanoparticles, termed OC47-Ce6, were engulfed by neutrophils in the inflammatory microenvironment following surgical tumor resection and subsequently trafficked along chemotactic gradients toward residual tumor sites (Guan et al., 2025). At there, OC47-Ce6 not only promotes the polarization of tumor-associated macrophages (TAMs) toward the pro-inflammatory M1 phenotype, but also blocks immunosuppressive signaling in tumor cells (Guan et al., 2025). More critically, upon exposure to near-infrared laser irradiation, Ce6 generated reactive oxygen species (ROS), inducing localized tumor cell death and promoting the release of tumor-associated antigens (Guan et al., 2025). Collectively, the integration of photodynamic therapy with the delivery of immunotherapeutic agents offers a valuable direction to enhance the efficacy of cancer immunotherapy.

Radiation-responsive drug delivery systems represent an emerging strategy for achieving precise, spatiotemporal control of therapeutic release at irradiated tumor sites. Recent studies have demonstrated the promise of integrating BEVs with such systems to enhance the efficacy of cancer radio-immunotherapy. In one example, OMVs derived from *Escherichia coli* were engineered to display CD47 antibodies on their surface (Feng et al., 2022). These OMVs not only leveraged their inherent PAMPs to engage TLRs on TAMs, but also targeted CD47-expressing tumor cells. This dual-binding mechanism facilitated the formation of a two-way adaptors between macrophages and tumor cells, promoting M1 polarization of TAMs and disrupting the CD47–SIRPα signaling axis to restore macrophage phagocytic activity (Feng et al., 2022). To further improve delivery specificity and safety, a diselenide- contained polyethylene glycol (PEG) shell was introduced to reduce immunogenicity during systemic circulation while enabling radiation-triggered release at tumor sites via cleavage of diselenide bonds (Feng et al., 2022). In another study, an ionizing radiation-inducible expression system was developed in *E. coli* Nissle 1917 (EcN) to enable targeted delivery of therapeutic proteins (Wang et al., 2025). Specifically, a fusion gene encoding a TREM2-specific single-chain variable fragment (scFv) was placed downstream of an SOS promoter (rAO) and integrated into EcN (Wang et al., 2025). Upon exposure to 8 Gy X-ray irradiation, the SOS response was activated, leading to high-level expression of scFv. Benefiting from the natural tumor-tropism of EcN and the enhanced permeability and retention (EPR) effect, scFv was packaged into OMVs and selectively delivered to hypoxic tumor regions, where it bound TREM2-expressing macrophages to alleviate immunosuppression (Wang et al., 2025). In general, the innovative application of radiation-responsive BEV-based delivery systems offers a novel paradigm for achieving precise and timely drug release within the context of tumor radiotherapy. Importantly, beyond their role as carriers, BEVs possess intrinsic immunogenic properties that enable them to actively reshape the immunosuppressive tumor microenvironment, an advantage that is difficult to replicate with conventional synthetic nanocarriers.

BEV-based delivery systems have also demonstrated significant potential in the development of novel antimicrobial strategies. By leveraging the modifiability of bacterial OMVs, several innovative approaches have been proposed to enhance both targeting specificity and antibacterial efficacy. Wei et al. designed OMVs targeting *Acinetobacter baumannii* by genetically modifying a msbB mutant *E. coli* strain and fusing the ClyA coding region with the targeting antibody fragment. Antimicrobial drugs coated with these OMVs exhibited exceptionally strong killing power against *A. baumannii* (Wei et al., 2024). In another study, Yang et al. developed a pH-responsive OMV-based system tailored to the acidic microenvironment of infection sites (Yang et al., 2025). They modified *E. coli*-derived OMVs with hydroxamate-type siderophores to direct vesicles toward *Staphylococcus aureus*, and further coated the OMVs with pH-sensitive calcium carbonate (CaCO₃) layers (Yang et al., 2025). Upon exposure to the acidic milieu of infected tissues, the CaCO₃ shell dissolved, triggering the release of encapsulated lysostaphin (Lsn) and mupirocin (Mup), which led to effective eradication of both intracellular and extracellular *S. aureus* (Yang et al., 2025). These studies collectively underscore the value of integrating microenvironment-responsive features of infection sites into BEV-based systems to achieve controlled and site-specific antimicrobial delivery. Future efforts should focus on the rational design of pathogen-specific targeting strategies, which exploit the distinct biological features of infectious agents to optimize the precision of BEV-mediated antimicrobial therapies.

The integration of gene-editing technologies such as CRISPR-Cas systems with BEV-based delivery platforms offers a transformative approach for precision medicine. Recent studies have demonstrated the feasibility of loading BEVs with CRISPR components for targeted genome editing. Jia et al. developed a hybrid nanoplatform by integrating OMVs with cationic lipids to deliver CRISPR/Cas9 plasmids targeting drug-resistant bacteria (Jia et al., 2024). In this system, OMVs provided inherent tropism toward antibiotic-resistant strains via surface protein interactions, while cationic lipids facilitated electrostatic complexation with negatively charged CRISPR/Cas9 plasmids and enhanced bacterial uptake (Jia et al., 2024). Upon delivery, the CRISPR/Cas9 plasmids induced lethal chromosomal damage, leading to the complete eradication of resistant pathogens (Jia et al., 2024). Similarly, Lin et al. constructed an oral micro nano system by encapsulating OMVs loaded with Cas9/sgRNA ribonucleoprotein (RNP) nanoclusters within pH-responsive calcium alginate microbeads (Lin et al., 2024). This design protected the cargo from gastric degradation and enabled targeted release in the intestine (Lin et al., 2024). Once released, the OMVs target to inflammatory macrophages and delivered Cas9 RNPs to disrupt TNF-α expression, resulting in marked alleviation of colitis symptoms in inflammatory bowel disease models (Lin et al., 2024). These studies collectively underscore the versatility of BEVs as delivery vehicles for nucleic acid therapeutics, particularly for precise genome editing in infectious and inflammatory diseases.

In addition to their role as drug delivery vectors, BEVs have also demonstrated considerable promise in cancer vaccine development due to their intrinsic immunogenicity and preferential uptake by antigen-presenting cells (APCs). For instance, Dong et al. encapsulated the model antigen ovalbumin into OMVs and achieved targeted delivery to dendritic cells (DCs), resulting in potent activation of antigen-specific immune responses and enhanced antitumor immunity (Dong et al., 2024).

Several recent high-quality reviews have provided valuable insights into the evolving landscape of BEV research. Notably, De Langhe et al. conducted a comprehensive analysis of 845 BEV-related publications from 2015 to 2021, identifying best practices and key knowledge gaps that currently hinder the standardization and translational potential of BEV research (De Langhe et al., 2024). By systematically evaluating aspects such as BEV sources, isolation techniques, storage conditions, characterization methods, and overall research transparency, the authors proposed targeted recommendations (De Langhe et al., 2024). These include unifying nomenclature and terminology, expanding investigations into BEVs naturally present in biological fluids such as blood, urine, and feces, optimizing characterization techniques, standardizing storage protocols, and improving the reproducibility and transparency of reporting (De Langhe et al., 2024). In another review, Liu et al. offered a detailed summary of current methodologies for BEV extraction and analysis, emphasizing their diagnostic potential from biological fluids and therapeutic applications in vaccines, cancer treatment, and tissue regeneration (Liu et al., 2024).

In general, BEVs remain emerging nanocarriers in the field of drug delivery. Owing to their superior targeting ability and unique immunogenicity, BEVs present a promising pathway for precise and efficient drug delivery.

##### 4.2.3.2 BEVs and cancer

While BEVs are increasingly utilized as drug delivery platforms in various disease contexts, their unique immunomodulatory capacities in cancer therapy warrant dedicated discussion. Therefore, this section focuses on the therapeutic potential of BEVs as active agents in reshaping the tumor microenvironment and eliciting antitumor immunity. In 2017, Kim and colleagues were the first to apply OMVs for tumor treatment. Their study demonstrated that OMVs exhibited a remarkable ability to induce long-term antitumor immune responses through the activation of the interferon-γ response, which could completely eradicate established tumors (Kim et al., 2017). This study highlights the potential of BEVs as tumor immunotherapeutic agents and provides valuable insights into combating tumors. In the following years, BEVs have made rapid advances in activating antitumor immune responses as potent immunostimulants, utilizing their inherited molecular patterns from parental bacteria. Park KS et al. synthesized BEVs that inhibit melanoma growth by inducing Th1 polarization. Moreover, synergistic use with anti-PD-1 inhibitors enhances immunotherapeutic efficacy (Park et al., 2021). The rapid accumulation of the extracellular matrix (ECM) in desmoplastic solid tumors generates physiological barriers that impede immune cell infiltration and the delivery of anticancer drugs. A tumor-targeting bacterial system based on *Escherichia coli* Nissle, which can reprogram tumor mechanisms and enhance immunotherapeutic efficacy through the release of cytolysin A (ClyA)-hyaluronidase (Hy) by OMVs, has been developed (Thomas et al., 2022). Additionally, the hypothesis that OMVs enhance antitumor immunity by triggering acute inflammation has been suggested (Xu et al., 2024).

The immunosuppressive tumor microenvironment (TME) in solid tumors often impedes the efficacy of immunotherapy, and BEVs have been shown to be effective in stimulating TME-programmed rearrangements to increase antitumor efficacy (Qing et al., 2020). Recent studies have highlighted the potential of leveraging the intrinsic immunogenicity of BEVs to enhance antitumor immune responses. Lu et al. designed a nano-vaccine by decorating OMVs from *E. coli* with PD-L1 antibodies to enhance tumor antigen presentation and disrupt immune checkpoint signaling simultaneously (Lu et al., 2025). While delivering tumor-specific antigens, the OMVs activated co-stimulatory second signals in APCs, and the anti-PD-L1 antibodies effectively blocked inhibitory PD-L1 signaling within the tumor, thereby enabling robust activation of tumor-specific immune responses (Lu et al., 2025). In a similar vein, Yao et al. developed a dual-drug delivery platform by camouflaging ascorbic acid and bortezomib-loaded nanoparticles with *E. coli*-derived OMVs (Yao et al., 2024). Upon release at the tumor site, the immunogenic OMV shell promoted DCs maturation and increased intratumoral infiltration of cytotoxic T lymphocytes (CTLs), highlighting the capacity of BEVs to reverse the immunosuppressive milieu of tumors (Yao et al., 2024).

Beyond enhancing adaptive immune responses, BEVs have also been explored as tools to induce immunogenic cell death (ICD) via pyroptosis, further reshaping the TME toward an inflamed phenotype. Li et al. developed a pyroptosis-inducing agent based on *E. coli*-derived OMVs that triggered caspase-11 activation and GSDMD cleavage following tumor cell internalization, culminating in inflammatory pyroptotic death and subsequent DCs maturation and CD8 + T cell activation (Li et al., 2025). Complementarily, Wu M et al. engineered a bioinspired copper nanozyme (Cu₂O-OMV) by assembling Cu₂O nanoparticles with OMVs (Wu et al., 2025). In this platform, LPS embedded in OMVs activated pyroptosis through the non-canonical inflammasome pathway, while Cu₂O simultaneously induced Cuptosis (Wu et al., 2025). The combination of two distinct modes of ICD triggered extensive antigen release, DCs maturation, and CTL infiltration, demonstrating a potent synergistic antitumor effect.

Moreover, BEVs have emerged as attractive platforms to activate the STING pathway. A recent study reported the construction of cadherin-17-targeted OMVs, derived from *E. coli* MG1655 and loaded with a photoimmunotherapy agent (Xia et al., 2025). Upon tumor accumulation, bacterial dsDNA cargo within OMVs directly activated the cGAS-STING axis in tumor cells, inducing ICD (Xia et al., 2025). Subsequent irradiation further amplified ICD via ROS production, resulting in massive endogenous dsDNA release and extensively activation of STING signaling in TAMs, ultimately enhancing antitumor immune responses (Xia et al., 2025).

In addition to these synthetic approaches, OMVs derived from commensal bacteria have shown promise in modulating immunosuppressive signaling pathways. For example, Zhu et al. demonstrated that *Akkermansia muciniphila*-derived OMVs, especially via the key protein Amuc_1434, downregulated PD-L1 expression in colorectal cancer cells (Zhu et al., 2025). This blockade of the PD-1/PD-L1 axis restored CD8 + T cell function, as evidenced by increased proliferation and elevated secretion of IFN-γ and IL-2, contributing to tumor regression and TME remodeling (Zhu et al., 2025).

Combining BEVs with photothermal therapy (PTT) offers another promising pathway to overcome the immunosuppressive TME (Li et al., 2022; Zhuang et al., 2022). PD-L1 antibody-modified attenuated *Salmonella* OMVs encapsulated with photosensitizers can activate DCs and CD8 + T cells to modulate immunity (Zhang et al., 2022).

In summary, the therapeutic potential of BEVs in modulating antitumor immunity and reprogramming the TME is being increasingly explored and refined. The integration of BEVs’ intrinsic immunogenicity and versatile editability with advanced bioengineering approaches is profoundly advancing the development of cancer immunotherapeutic strategies. Moreover, emerging technologies such as single-cell sequencing offer powerful analytical tools to further elucidate the complex immunological mechanisms underlying BEV-based therapies. By enabling high-resolution profiling of heterogeneous immune cell populations within the TME, single-cell sequencing can help identify key cellular targets and track dynamic immune responses following BEV administration. In the context of personalized medicine, single-cell profiling of patient-specific TME features may further guide the rational design of individualized BEV formulations optimized for maximum therapeutic efficacy.

### 4.3 Remarks and future perspectives

Over the past two decades, research on BEVs has undergone remarkable growth, evolving from an emerging niche into a rapidly maturing field. The exponential increase in publication output and the expansion of global collaboration networks reflect both the scientific community’s enthusiasm and the perceived translational potential of BEVs. Initially centered on elucidating their biological characteristics, the research focus has progressively shifted toward leveraging BEVs as multifunctional nanoplatforms for therapeutic delivery and immune modulation. Nonetheless, ongoing discoveries regarding the multifaceted roles of BEVs in microbiota–host interactions, continue to shape and expand the field’s frontiers.

Through a comprehensive bibliometric analysis, this study has provided valuable insights into the current research landscape and anticipated directions of BEV development. Engineered BEV-based drug delivery systems have emerged as a major focal point, transitioning from basic drug carriers to sophisticated, multimodal therapeutic platforms integrating advanced bioengineering techniques. Future innovations are likely to center on combinatorial approaches, including co-delivery with immune cells, modular surface modifications for precise targeting, and stimuli-responsive release mechanisms, such as pH, light, ultrasound, or radiation triggers, that enhance therapeutic precision and control. Moreover, the intrinsic high immunogenicity of BEVs renders them particularly attractive as promising candidates for cancer immunotherapy and TME remodeling, both of which constitute key directions for future exploration and application. Synergizing BEVs with immune checkpoint inhibitors and other immunotherapeutic agents holds the potential to overcome immunosuppressive barriers that limit the efficacy of conventional treatments. To facilitate clinical translation, future studies are warranted to further elucidate the mechanisms through which BEVs enhance antitumor immune responses, inhibit immunosuppressive signaling in tumor cells, and induce ICD. Ongoing progress in nanotechnology, biomedical engineering, and industrial-scale biomanufacturing is expected to empower BEVs with the capacity to address long-standing clinical challenges through innovative therapeutic strategies.

### 4.4 Limitations

In this study, innovative bibliometric methods were utilized to analyze publications on BEVs comprehensively. However, it is necessary to acknowledge some limitations of this study. First, the data for this study were obtained exclusively from WOSCC, and some publications from other databases may have been omitted. Although WOSCC offers high-quality literature, comprehensive multidisciplinary coverage, robust citation tracking, and standardized data formats that are particularly well-suited for bibliometric analysis, the potential database bias should be acknowledged and carefully considered. Second, this study included only English-language publications, which may introduce a degree of language bias. Future research should aim to incorporate multiple databases and include publications in various languages to enable a more comprehensive and globally representative assessment of BEV-related literature. Furthermore, the R package “Bibliometric” and CiteSpace software cannot analyze the full text of a publication, which may result in some details being omitted. Finally, publications indexed to the WOSCC after the date of our search were not included, which may have resulted in missing the latest research advances in the field. Future studies should evaluate publications from other databases, and more accurate bibliometric methods should be developed.

## 5 Conclusion

This study provides a comprehensive bibliometric analysis of publications on BEVs. By objectively examining international collaboration, output distribution, research status, and research hotspots and predicting future development trends, the results provide valuable insights into the current field and inspire new ideas. Our study highlights that BEV-based drug delivery systems are a major focus of current research, with tremendous potential. Significant progress has been made in the use of BEVs for targeted tumor therapy, antimicrobial applications, and nanovaccines. Future research on BEVs is expected to focus on tumor immunotherapies and remodeling of the tumor immune microenvironment. With the advancements in nanotechnology, biomedicine, and industry, BEVs are anticipated to make remarkable strides and greatly contribute to solving challenging clinical issues.

## Data Availability

The original contributions presented in the study are included in the article/supplementary material, further inquiries can be directed to the corresponding author.
